# Sustaining efforts to improve family well-being with parents with mental ill health and substance (mis)use

**DOI:** 10.3389/fpsyt.2024.1376409

**Published:** 2024-03-25

**Authors:** Becca Allchin, Kurt Albermann, Kate Blake-Holmes, Lina Gatsou, Rochelle Hine, Karin van Doesum, Joanne Nicholson

**Affiliations:** ^1^ Mental Health Program, Eastern Health, Melbourne, VIC, Australia; ^2^ School of Rural Health, Faculty of Medicine, Nursing & Health Sciences, Monash University, Clayton, VIC, Australia; ^3^ Social Pediatric Center, Cantonal Hospital, Winterthur, Switzerland; ^4^ School of Social Work, Faculty of Social Sciences, University of East Anglia, Norwich, United Kingdom; ^5^ Institute of Health, Health Policy and Social Care Research, De Montfort University, Leicester, United Kingdom; ^6^ Child and Adolescent Mental Health Services, Leicestershire Partnership NHS (National Health Service) Trust, Leicester, United Kingdom; ^7^ Monash Rural Health, Monash University, Warragul, VIC, Australia; ^8^ Department of Clinical Psychology, Radboud University Nijmegen, Nijmegen, Netherlands; ^9^ Community Mental Health Service, Department Impluz Prevention, Dimence-groep, Deventer, Netherlands; ^10^ The Heller School, Institute for Behavioral Health, Brandeis University, Waltham, MA, United States

**Keywords:** sustainability, implementation, family mental health, parents with mental ill health and/or substance (mis)use, children of parents with mental ill health

## Abstract

Research conducted over the past 30 years has developed an extensive body of knowledge on families where parents experience mental ill health and/or substance (mis)use, and interventions that are effective in improving their outcomes. A more recent focus has also explored the importance and nuance of implementation. This perspective article reflects on the concept and practice of sustainability within this body of work and considers underlying assumptions in the field about the goal and direction of interventions that make clarity about sustainability difficult. We identify challenges for understanding sustainability, relating to how and who defines it, what is measured and the impact of context. We conclude by considering how we might be better able to plan and design for sustainability within this field.

## Introduction

A vast body of work has been built globally aimed to improve the wellbeing of all family members when a parent experiences mental ill health, and/or substance (mis)use (1). Over 30 years, the field has built knowledge of the population (2–4) and developed and tested an array of interventions designed for different settings and populations (5–7). Collections of papers in edited volumes and journal special issues have brought attention to the issues and the range of global research efforts, while documenting shifts and progress over time (1, 8–12).

A strong presence in scientific literature cannot be presumed to reflect a presence in real world settings, however, and a gap between research and practice is evident in this field. As the global conversation has identified the intricacies involved in promoting and improving wellbeing and translating research to practice, there have been shifts in the research from standalone intervention effectiveness and efficacy studies to studies that explore the influence of context and implementation (13–16). Studies on sustainability remain uncommon, and so it is important to reflect on what sustainability means in this field and what factors need to be considered.

Sustainability is interrelated with other concepts of implementation. The many frameworks, models and theories in implementation science have in common a multifactorial map of components (17–19). While an intervention’s characteristics such as its acceptability, fidelity and feasibility influence sustainability (20), the internal and external context and the capacity to sustain (e.g., funding resources, workforce) also shape outcomes (21).

Achieving sustainability in health and social care is complex and difficult, with a recent review finding that sustainability is rarely achieved (20). A lack of clarity of, and consensus on the definition of sustainability and how it is measured contributes to this difficulty (22, 23). Within the literature on implementation science, a confusing array of terminology is used for sustainability derived from the different fields of study (24). Two views guide sustainability approaches; i) sustainability as the end point of a linear process and, ii) sustainability as a process that needs attention to promote long term impact (23). These two approaches lead to very different ideas about how success is understood and measured.

While a common definition of sustainability is the continuation and/or maintenance of a program or its activities, this can lead to incomplete and potentially misleading results as activities can be continued without the desired outcomes being delivered (21, 23). Instead, a definition that considers the interrelated nature of concepts underpinning sustainability is recommended. For example Moore et al. (22)’s definition includes continued delivery (with or without adaptation) after a defined time that produces continued benefits. Scheirer and Dearing (25) suggest three layers of indicators are needed to understand sustainability fully: continued benefits, continued practice, and continued capacity to practice.

This paper is the reflections of the Sustainability and Spread Working Group of the *International Research Collaborative for Change in Parent and Child Mental Health*, a collective of international experts involved in research and practice in the field. The article aims to clarify and define the concepts of sustainability as applied to this body of work and to raise questions to support the field as it moves forward.

## Definitional challenges

Definitional challenges make the study and practice of sustainability difficult. While it may seem easy to measure sustainability as whether a policy or practice is still there or not, understanding the purpose (i.e., what we want to sustain and why) is key. In this field of study there has been a focus on changing practice, developing, and implementing interventions, and the broader work of changing cultures and systems. None of these are ends in themselves but are means to bring about transformational change that will have ongoing beneficial outcomes for children, parents, and families where parents experience mental health challenges. While measuring the maintenance of a changed policy or practice or an intervention’s use may seem helpful, unless we also know if this activity is meeting the intended purpose, we may be celebrating the sustaining of an activity without knowing if its impact is beneficial, benign, or even harmful.

Take for example the focus on changing the practice of identifying parents with mental ill health and their children. Identification has been heralded as an important first step for enabling prevention and providing support, resulting in calls for systems of identification (26–28). However, studies of the implementation of identification processes in Norway and The Netherlands, highlight that identification may not lead to these outcome (29, 30). Further, Everts et al. (29) suggest that a risk and reporting lens to identification distracted practitioners from identification leading to providing support to children. Identification in and of itself may be sustained but could lead to increased surveillance and stigmatisation of families and disruption to the collaborative, strength-based approaches needed to engage with and support families.

Being clearer about the core functions and understanding the core mechanisms of change of an intervention or practice can help in the quest for sustainability. An intervention’s *core function* is its intended purpose – why it matters, while the *form* of intervention is its activities - what is done, by whom and when, where and how it is carried out. Depending on the execution of the activities, they may or may not fulfil the intended core functions (31). For example, delivering Let’s Talk about Children (LTC) as a checklist to assess a child’s development and refer to external agencies, is unlikely to result in meeting its core function of engaging parents in a partnership to promote child wellbeing.

How or why the intervention’s activities (core forms) achieve its purposes (core functions) is described as its *mechanisms of change* (31–33). Mechanisms of change are theory driven reasons for change (34, 35). For example Goodyear et al. (36) suggested that LTC’s activities enabled parents to build new perspectives on themselves and their parenting (mechanism for change) that enhanced parent’s agency and self-efficacy to promote their child’s wellbeing (core function). More clearly identifying the core functions and mechanisms for change for interventions, enables training materials and resources to make the functions and mechanisms of change overt, thus increasing understanding of how to measure and sustain what is integral to achieving the intended outcome (34, 35, 37). This will require working to articulate the principles, theory and assumptions underpinning an intervention, the need it is attempting to address and the change or outcome it hopes to achieve.

It is challenging, in this broad field, to have clarity about the outcomes we want to see and for whom (38). There are decades of studies exploring the needs from different perspectives: parents, family members, practitioners, service systems, researchers. However, how these needs are identified, explored and framed impact the solutions generated, the outcomes hoped for and, in turn, how sustainability is measured and understood. For example, an assumption of the workforce’s needs for support to confidently and capably deliver family-focused practice might lead to the development of an intervention of practice support consultations (39). Sustainability might be seen as the continuation of the consultations. The deeper need, however, is for families to receive family-focused practice, which is dependent on the workforce’s ability to confidently and capably deliver family-focused practice. The sustaining of the intervention, in this instance defined as the workforce consultations, may be essential or irrelevant to either of these two outcomes. Isobel et al. (38) notes that within a range of outcomes envisioned there is globally a broad commitment to “*improving outcomes for children, parents and families*”. They argue for developing shared outcomes defined by those for whom the outcomes matter, to support the tracking of the sustained changes needed on a global scale (38).

There are many layers to the outcomes of prevention of harm and promotion of wellbeing for all family members – adults and children. No single intervention or implemented change of practice will meet such broad overall outcomes. Instead, the work requires a suite of practices from identification at service entry, care coordination, service system navigation, skill-building practices, therapeutic engagement, community connection, advocacy, and many others depending on the family’s identified needs and available socioeconomic resources, and access to service options and supports. The success or sustainability of one practice might also be undermined by a lack in other practices. For example, the success of workforce training regarding the needs of families might be seen in the practitioner’s increased awareness of their adult service-user’s parental status. However, without the skills, confidence and competence of the workforce and organisational capacity to support practitioners’ practice, there may be no tangible difference in family wellbeing (40, 41). Understanding the complexity required to make meaningful responses to promote the wellbeing of all family members allows for acknowledging the interconnection among practices and to keep in view the overall goal while measuring the sustainability of a part.

Keeping in mind Scheirer and Dearing (25) definition of sustainability noted above, the sustainability of a practice needs to be held in the light of a continuation of the expected benefit and the continuation of the organisational level support required to practice. The continuation of a practice such as the use of the Child Check, while enabling continued identification, might not result in the continuation of the expected benefit of support to children (29). A lack of support for practice, such as no system to allocate parents to trained practitioners, might impact the practitioner’s ability to stay fluent in the new practice (42). To measure what is sustained, we need to find ways to take these complexities into account, to be able to tell a clear story of sustainability.

## Context challenges

Consideration of context on macro, meso, and micro levels is pivotal to understanding sustainability. At the macro level (nations, states, provinces or territories), structures and systems shape the way organisations are set up, the composition of their workforce, and the type of work that is funded and mandated. An intervention that does not fit current priorities, the funding model or role of workforce is unlikely to be sustained without the long-term investment required to create change at this level. Macro level shifts in policy or mandates can be important to facilitate sustained change but have little power without the engagement of the meso-level delivery organisations. It is at this level, made up of public, private and charitable organisations, that infrastructure is build and adjustments are made to enable new practice to be prioritised, integrated, delivered and sustained in practice. At the micro level are the actions of individuals (practitioners, families, leaders, et al.) who deliver, receive or support and provide governance for these new practices and can influence its sustainability. Focusing on each of these levels is important to the story of sustainability.

Health, education, social and community contexts are dynamic environments with moving parts at each level effecting other levels, making sustainability difficult to understand. As a new practice is being implemented, there may be shifts in government policy effecting how organisations work and who or what is prioritised. Organisations may have shifts in leadership or policy that impact support, work schedule, or who is deemed to be a priority or target population. Individual practitioners may change roles or organisations, taking their new skills, knowledge and support out of the organisation. The “new” context may require adaptation of the intervention to make it fit the setting with its particular workflow, clinical guidelines and/or specific target population. Measures of services received, training numbers or continuity, continued practice, or organisational supports will all give an incomplete picture. Sustainability needs to be able to be measured or understood within the realty of the dynamic world it inhabits (43) and with a focus on the operationalisation of the intended change mechanisms.

Furthermore, interventions don’t stand alone but exist within a broader context of service systems and other interventions that influence sustainability. Take for example Child Talks in The Netherlands and LTC in Finland, which were developed *in situ* and sit within a suite of structures and interventions (44–46). These context-related factors provide (or inhibit) entry points to the intervention, authorising systems for its use, and linkages to further support if required, and work as scaffolding around the interventions, influencing their sustainability. Focussing narrowly on one intervention without this broader context might overlook pivotal components of its sustainability.

## Measurement challenges

The matrix of outcomes needed to measure sustainability adds to the difficulty of understanding sustainability. Simple measures of fidelity of practice against manualised activities might miss the core function being sustained despite the adaptations made to its form to fit the changing settings or target populations. Measures of endpoint use might miss the reality of the workforce changes between time 1 and 2. The need to collect the three levels of outcomes – benefits, practice, and capacity to support practice (25) - makes measuring sustainability complex and time consuming. Lauritzen and Reedtz (19) suggest that creating sustainable practices requires working in closer partnership with practice settings over longer periods of time than traditional research projects, a more resource intensive undertaking.

This raises another challenge for understanding sustainability. Much of the work of understanding the need and developing innovations and interventions in this field is known through the publication of research findings in scientific journals. The context of the research world is driven by (what is often) politically-motivated project-based grant funding that dictate time limited engagement. While this may be well suited to the development and testing of innovations, sustainability or its lack happens within practice settings that dance to a different tune (47). Allchin et al. (47) suggests that practice settings need to be equipped to utilise implementation science to support the monitoring and adaptation needed for sustainability.

## Designing for sustainability

The shifts required to create sustained systems and practices that enable better outcomes for parents, children and families are complex. Additionally, at the heart of this work is the understanding that no two families are alike, and that the outcomes prioritized by one family may not be the same as those prioritized by another. It may be, for example, that the achievement of goals is a better indicator of success than a variable that stands as a proxy for improvements in wellbeing or reductions in symptoms. Embracing the complexity is necessary to create lasting change that promotes the wellbeing of parents, children and families. In highlighting challenges for understanding and therefore supporting sustainability in this field, this paper creates a foundation for further exploration and proactive planning in the design, delivery, and evaluation of interventions.

As we move forward, it is clear that for parents, children and families to benefit from what is known to be effective, we need to embed sustainability thinking into the work from the outset (48). When developing interventions, the core functions and mechanisms of change need to be articulated in a way that allows implementors to facilitate practice settings to embed them in routine monitoring systems and practitioners to use interventions flexibly to achieve them. A matrix of outcome measures to capture the three levels - benefits for families, practitioners’ practice, and capacity to support practice - needs to be considered as part of implementation. Measures must be meaningful and relevant to the families, practitioners and organizations engaged in services. These measures need to be efficient and cost effectively so as to be able to be used by practice settings in everyday practice. The implementation process needs to have feedback loops that monitor and adjust efforts over time to support sustained practice. Extending implementation models in this way provides a framework for research into sustainability. An example of this is seen in the model of sustainability of family focused practice in adult mental health services (47) which outlines the interconnected micro, meso and macro level outcomes to support measurement for both research and practice.

We need to consider how the individual pieces of work contribute to the bigger story of better outcomes. What are the shared outcomes we are working towards? What role does our contribution play? Who determines what is important to sustain? What sorts of measures allow us to describe and discuss sustainability as part of the bigger intervention development, initial implementation, and testing story? What would it take for research and practice settings to work in closer partnership over the longer periods of time needed to tell it?

## Conclusion

This paper brings to light the critical conversation about sustainability in the field focused on improving outcomes for families where parents experience mental ill health and/or substance (mis)use. Sustainability is difficult to understand, measure and work towards due to its definitional challenges and the constant change at all levels in real-world settings. The fit between the setting and its needs, and the intervention and its purpose, requires a clearer attention to and articulation of intervention’s core functions (purpose) and mechanisms of change (how change happens). Distilling these core functions and change mechanisms, as well as tracking their input to improving outcomes, is a vital contribution to the literature. Knowing these core functions and change mechanisms will enable implementers and practitioners to make the adaptations of the activities to fit settings without compromising outcomes for families and equip managers to know how to support and monitor for the core functions in their changing circumstances. How we move towards sustainability is important. Researchers, implementers, managers, practitioners and families need to partner from the very beginning of the development and implementation process to design for sustainability over time through embracing complexity, defining our shared visions and outcomes, and identifying the contribution of our part to the bigger story.

## Data availability statement

The original contributions presented in the study are included in the article/supplementary material. Further inquiries can be directed to the corresponding author.

## Author contributions

BA: Writing – review & editing, Writing – original draft, Project administration, Conceptualization. KA: Writing – review & editing, Writing – original draft. KB-H: Writing – review & editing, Writing – original draft. LG: Writing – review & editing, Writing – original draft. RH: Writing – review & editing, Writing – original draft. KD: Writing – review & editing, Writing – original draft. JN: Writing – review & editing, Writing – original draft.
